# Prognostic Impact of Genetic Polymorphism in Mineralocorticoid Receptor and Comorbidity With Hypertension in Androgen-Deprivation Therapy

**DOI:** 10.3389/fonc.2018.00635

**Published:** 2018-12-18

**Authors:** Masaki Shiota, Naohiro Fujimoto, Kenjiro Imada, Eiji Kashiwagi, Ario Takeuchi, Junichi Inokuchi, Katsunori Tatsugami, Shunichi Kajioka, Takeshi Uchiumi, Masatoshi Eto

**Affiliations:** ^1^Department of Urology, Graduate School of Medical Sciences, Kyushu University, Fukuoka, Japan; ^2^Department of Urology, School of Medicine, University of Occupational and Environmental Health, Kitakyushu, Japan; ^3^Department of Clinical Chemistry and Laboratory Medicine, Graduate School of Medical Sciences, Kyushu University, Fukuoka, Japan

**Keywords:** androgen-deprivation therapy, castration resistance, hypertension, mineralocorticoid receptor, prostate cancer

## Abstract

Mineralocorticoid receptor (MR) signaling which is closely associated with hypertension plays important roles in resistance to antiandrogen therapy in prostate cancer. However, its impact on the prognosis in androgen-deprivation therapy (ADT) has not been elucidated. Then, we investigated the impact of genetic variation in MR and comorbidity with hypertension on the prognosis in ADT. This study included 182 Japanese patients with prostate cancer treated with ADT, whose comorbidity status with hypertension were available. The associations of MR polymorphism (rs5522) and comorbidity with hypertension with clinicopathological parameters as well as progression-free survival and overall survival were examined. Clinicopathological characteristics were comparable between genetic variation in MR. However, homozygous variant in MR was associated with shorter time to castration resistance (*P* = 0.014) and any-cause death (*P* = 0.024). In patients' background, presence of comorbidity with hypertension showed the trend with lower PSA level at diagnosis and lower biopsy Gleason score, as well as significant association with less incidence of N1. Comorbidity with hypertension was associated with longer time to castration resistance (*P* = 0.043) and any-cause death (*P* = 0.046), which was diminished on multivariate analysis including age, PSA level at diagnosis, biopsy Gleason score, clinical stage, and the modality of hormonal therapy. Genetic variation in *MR* (rs5522) and comorbidity with hypertension were significantly and potentially associated with prognosis when treated with ADT, respectively. This suggests that the individual intensity of MR signaling may be associated with resistance to ADT and a promising biomarker in ADT.

## Introduction

Androgen-deprivation therapy (ADT) is one of standard pharmacotherapies for prostate cancer (1). Although ADT is usually effective at the initial stage, most patients show resistance to ADT, and develop castration-resistant prostate cancer (CRPC). So far, various mechanism has been reported to contribute to obtaining castration resistance (2). Among them, aberrant signaling related to androgen receptor (AR) is thought to play critical roles in castration resistance (2, 3). In addition, glucocorticoid receptor signaling has recently been shown to play a key role in enzalutamide resistance as well as castration resistance by hijacking AR function, because steroid receptor including glucocorticoid receptor binds to the same sequence as AR, and can modulate the expression of AR target genes (4, 5). In addition, we have recently shown that another steroid receptor mineralocorticoid receptor (MR) signaling is involved in resistance to antiandrogen agent enzalutamide (6).

Recently, up-front pharmacotherapies utilizing docetaxel and abiraterone have been proved to be life prolonging in phase 3 clinical trials (1, 7). Accordingly, the risk stratification of the prognosis when treated with ADT is becoming more important to choose appropriate therapy for patients. So far, several genetic variations were reported to be associated with the prognosis in ADT (8). However, the association between genetic variation in MR signaling and the prognosis in ADT has not been investigated. In addition, MR signaling is shown to be associated with the comorbidity with hypertension (9), suggesting the interaction between MR signaling and hypertension may be involved in prostate cancer pathogenesis. Actually, comorbidity with hypertension was shown to be associated with increased risk of prostate cancer incidence by meta-analysis (10). However, the association between hypertension and the prognosis in ADT has not been reported. Then, in this study, we examined the prognostic impact of genetic variation in *MR* which causes missense mutation in Ile180Val, as well as comorbidity with hypertension among men treated with ADT.

## Patients and Methods

### Patients

Japanese patients who had undergone ADT with surgical castration or medical castration using a gonadotropin-releasing hormone agonist (goserelin acetate or leuprorelin acetate) and/or an antiandrogen agent (bicalutamide, flutamide, or chlormadinone acetate) for hormone-sensitive prostate cancer at Kyushu University Hospital and the University of Occupational and Environmental Health between 1993 and 2005 were included (11). Among these, only patients with available information on comorbidity with hypertension were included. Comorbidity with hypertension was defined as the use of anti-hypertensive drugs. Written informed consent was obtained from all patients. This study was performed in accordance with the principles described in the Declaration of Helsinki and the Ethical Guidelines for Epidemiological Research enacted by the Japanese Government and approved by each institutional review board.

All patients were histopathologically diagnosed with adenocarcinoma of the prostate. Clinical TNM staging was determined in accordance with the unified TNM criteria based on the results of digital rectal examination, transrectal ultrasound, magnetic resonance imaging, computed tomography, and bone scan (12). Progressive disease was defined as an increase in serum prostate-specific antigen (PSA) levels of >2 ng/mL and a 25% increase over the nadir, the appearance of a new lesion, or the progression of one or more known lesions classified according to the Response Evaluation Criteria in Solid Tumors (13).

### Polymorphism Genotyping

*MR* (rs5522) genotyping was performed as described previously (11, 14). Briefly, genomic DNA was extracted from whole blood samples. Genotyping was performed on a CFX Connect Real-Time System with pre-designed TaqMan SNP Genotyping Assays (Thermo Fisher Scientific) for *MR* rs5522 (C__12007869_20) and TaqMan Gene Expression Master Mix according to the manufacturer's protocol.

### Statistical Analysis

All statistical analyses were performed using JMP13 software (SAS Institute, Cary, NC, USA). Continuous and categorical data were analyzed by Wilcoxon rank sum and Pearson's chi square tests, respectively. The log-rank test was used to analyze survival between groups. Survival curve was determined by the Kaplan–Meier method. Cox proportional hazards model was used to estimate hazard ratios (HRs). All *p*-values are two-sided. Levels of statistical significance were set at *P* < 0.05.

## Results

The clinical and pathological characteristics of 182 Japanese patients included in this study are shown in Table 1. During median follow-up of 4.9 years (interquartile range, IQR; 2.2-7.3 years), 95 men (52.2%) and 68 men (37.4%) experienced progression to CRPC and any-cause death, respectively. We investigated the impact of single-nucleotide polymorphism in *MR* rs5522. Genotyping revealed the distribution of homozygous wild-type (AA), heterozygous variant (AG), and homozygous variant (GG) to be 119 (65.4%), 56 (30.8%) and 7 (3.9%) men, respectively. Patient characteristics including comorbidity with hypertension were comparable among patients with homozygous wild-type (AA), heterozygous variant (AG), and homozygous variant (GG) (Table 1).

**Table 1:** Patient characteristics among patients undergone ADT stratified by *MR* rs5522 polymorphism.

	**All**	***MR*** **polymorphism (rs5522)**		
	***n* = 182**	**AA (*n* = 119)**	**AG (*n* = 56)**	**GG (*n* = 7)**	***P*-value**
Median age, years (IQR)	71 (66–75)	72 (66–76)	71 (63–74)	71 (66–73)	0.40
Median PSA at diagnosis, ng/ml (IQR)	68.0 (13.9–298.1)	55.1 (12.7–263.5)	62.0 (17.8–600.0)	75.9 (12.1–276.2)	0.53
NA	3	1	2	0	
**BIOPSY GLEASON SCORE**, ***N*** **(%)**					
<7	24 (15.0%)	15 (14.6%)	8 (16.0%)	1 (14.3%)	
7	59 (36.9%)	42 (40.8%)	16 (32.0%)	1 (14.3%)	
≥8	77 (48.1%)	46 (44.7%)	26 (52.0%)	5 (71.4%)	0.53
NA	22	16	6	0	
**CLINICAL T–STAGE**, ***N*** **(%)**					
T1/2	69 (42.3%)	48 (46.2%)	19 (36.5%)	2 (28.6%)	
T3	68 (41.7%)	41 (39.4%)	23 (44.2%)	4 (57.1%)	
T4	26 (16.0%)	15 (14.4%)	10 (19.2%)	1 (14.3%)	0.70
NA	19	15	4	0	
**CLINICAL N-STAGE**, ***N*** **(%)**					
N0	119 (71.3%)	79 (73.8%)	36 (67.9%)	4 (57.1%)	
N1	48 (28.7%)	28 (26.2%)	17 (32.1%)	3 (42.9%)	0.53
NA	15	12	3	0	
**CLINICAL M-STAGE**, ***N*** **(%)**					
M0	90 (49.5%)	62 (52.1%)	27 (48.2%)	1 (14.3%)	
M1	92 (50.5%)	57 (47.9%)	29 (51.8%)	6 (85.7%)	0.12
**HORMONAL THERAPY**, ***N*** **(%)**					
Combined androgen blockade	109 (59.9%)	68 (57.1%)	36 (64.3%)	5 (71.4%)	
Castration	51 (28.0%)	37 (31.1%)	12 (21.4%)	2 (28.6%)	
Antiandrogen	22 (12.1%)	14 (11.8%)	8 (14.3%)	0 (0.0%)	0.44
**COMORBIDITY WITH HYPERTENSION**, ***N*** **(%)**					
Presence	89 (48.9%)	57 (47.9%)	29 (51.8%)	3 (42.9%)	
Absence	93 (51.1%)	62 (52.1%)	27 (48.2%)	4 (57.1%)	0.84

*IQR, interquartile range; NA, not available*.

**Figure 1:**
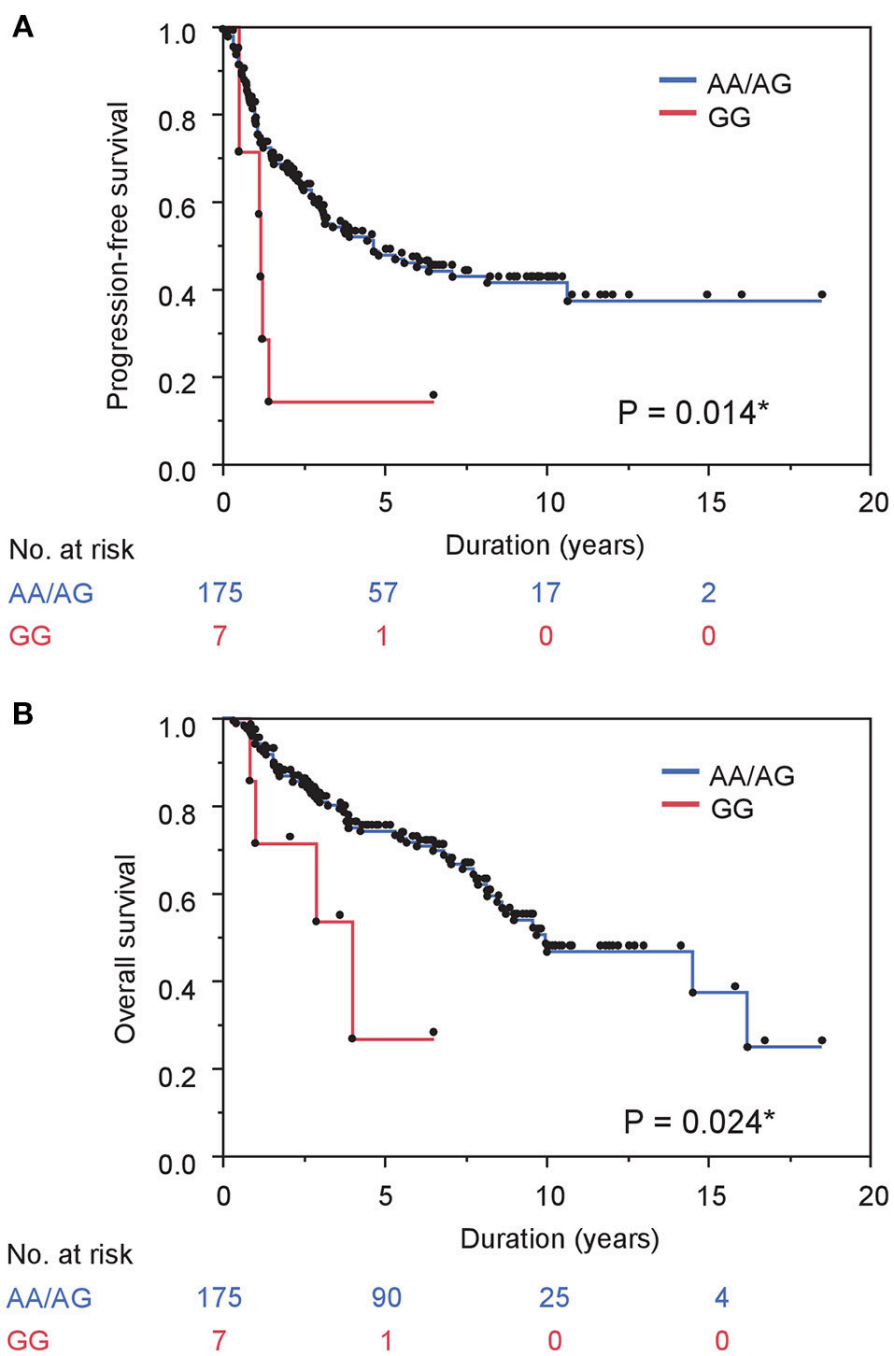
Gene polymorphism in *MR* rs5522 correlates with survival in prostate cancer cases treated with ADT. **(A)** and **(B)** PFS **(A)** and OS **(B)** stratified by gene polymorphism in *MR* rs5522 are shown.

The risk of both progression and any-cause death was higher in men carrying homozygous variant compared with those with homozygous or heterozygous wild-type allele while there were no differences in men with homozygous wild-type allele compared with those with heterozygous or homozygous variant (Table 2). A Kaplan–Meier curve shows that progression-free survival was significantly inferior in cases with homozygous variant (*P* = 0.014, Figure 1A). Similarly, the Kaplan–Meier curve shows significant inferior overall survival in cases with homozygous variant (*P* = 0.024, Figure 1B). On univariate analysis, homozygous variant showed significant and on borderline shorter time to castration resistance [hazard ratio (HR), 95% confidence interval (CI); 2.70 (1.05-5.72), P = 0.041] and any-cause death [HR (95% CI); 3.06 (0.92-7.57), *P* = 0.065], respectively (Table 2). Similarly, on multivariate analysis including age, PSA level at diagnosis, biopsy Gleason score, clinical stage, and the modality of hormonal therapy, homozygous variant (GG) was on borderline and significant independent prognostic factor of progression [HR (95% CI); 2.68 (0.98-6.19), P = 0.054] and any-cause death [HR (95% CI); 3.86 (1.08-10.78), *P* = 0.039], compared with those with a homozygous or heterozygous wild-type allele, respectively.

**Table 2:** PFS and OS among men undergone ADT stratified by *MR* rs5522 polymorphism, and comorbidity with hypertension.

**Variable**	**Secondary model (dominant)**			**Secondary model (recessive)**			**Comorbidity with hypertension**		
	**Major homozygote**	**1 or 2 minor allele HR (95% CI)**	***P*-value**	**1 or 2 major allele**	**Minor homozygote HR (95% CI)**	***P*-value**	**Absence of hypertension**	**Presence of hypertension HR (95% CI)**	***P*-value**
PFS	ref	1.07 (0.70–1.61)	0.75	ref	2.70 (1.05–5.72)	0.041^*^	ref	0.66 (0.44–0.99)	0.045^*^
OS	ref	1.30 (0.79–2.10)	0.29	ref	3.06 (0.92–7.57)	0.065	ref	0.61 (0.37–0.99)	0.046^*^

* *Statistically significant. PFS, progression-free survival; OS, overall survival; HR, hazard ratio; CI, confidence interval*.

**Figure 2:**
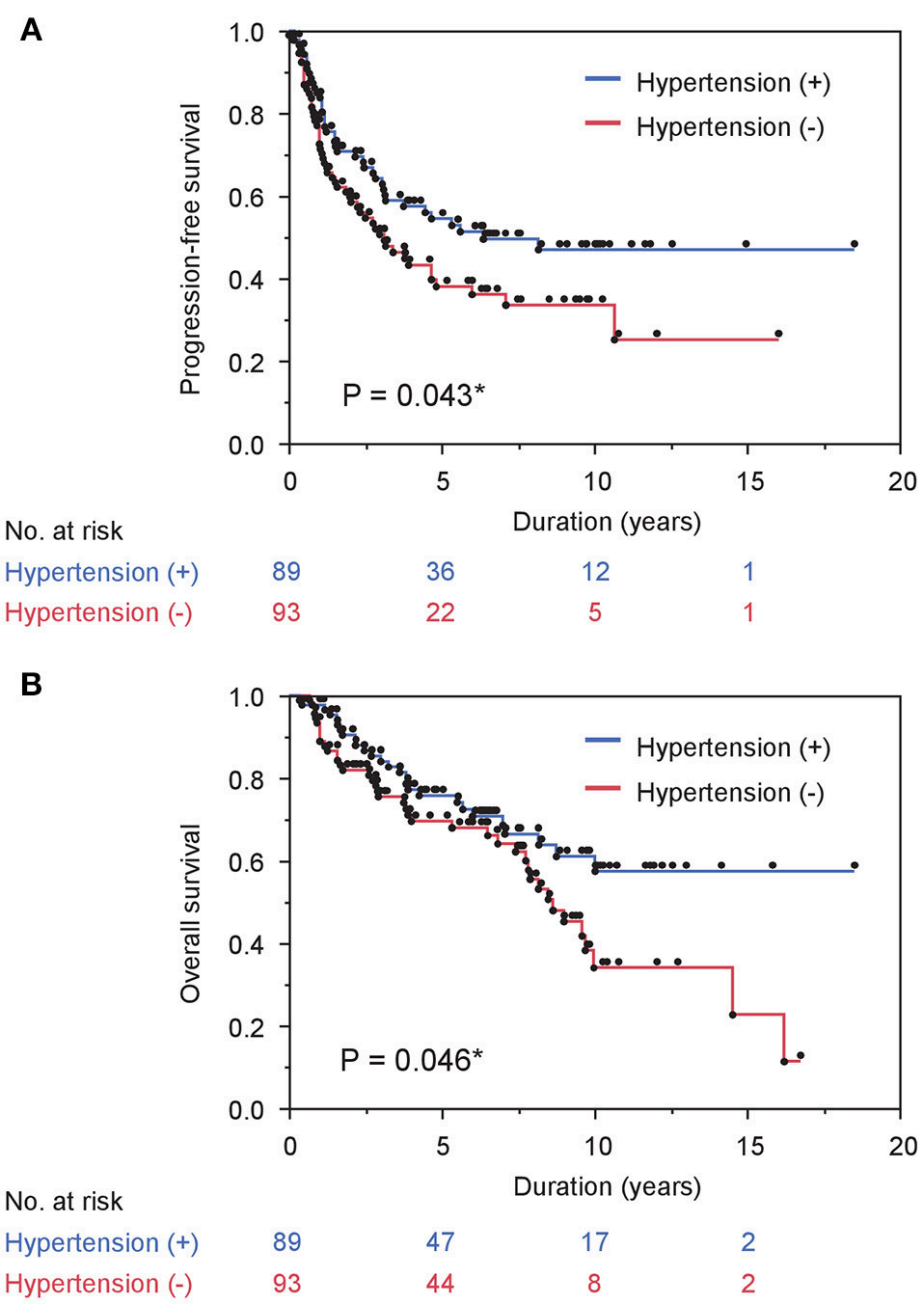
Comorbidity with hypertension correlates with survival in prostate cancer cases treated with ADT. **(A)** and **(B)** PFS **(A)** and OS **(B)** stratified by comorbidity with hypertension are shown.

Next, we investigated the association between comorbidity with hypertension and the prognosis of ADT in the same cohort. In patients' background, presence of comorbidity with hypertension showed the trend with lower PSA level at diagnosis and lower biopsy Gleason score, as well as significant association with less incidence of N1 (Table 3). As shown in Figure 2A, comorbidity with hypertension was associated with a lower risk of progression during ADT (*P* = 0.043). As well, surprisingly, comorbidity with hypertension was also associated with a lower risk of any-cause death (*P* = 0.046, Figure 2B). On univariate analysis, comorbidity with hypertension was associated with longer time to castration resistance [HR (95% CI); 0.66 (0.44-0.99), *P* = 0.045] and any-cause death [HR (95% CI); 0.61 (0.37-0.99), *P* = 0.046] (Table 2). However, on multivariate analysis including age, PSA level at diagnosis, biopsy Gleason score, clinical stage, and the modality of hormonal therapy, comorbidity with hypertension was not an independent prognostic factor of progression [HR (95% CI); 0.79 (0.47-1.31), *p* = 0.36] and any-cause death [HR (95% CI); 0.62 (0.33-1.17), *p* = 0.14], compared with absence of comorbidity with hypertension.

**Table 3:** Patient characteristics among men undergone ADT stratified by comorbidity with hypertension.

**Variable**	**Comorbidity with hypertension**		***P*-value**
	**Presence (*n* = 89)**	**Absence (*n* = 93)**	
Median age, years (IQR)	73 (66–75)	71 (64–75)	0.21
Median PSA at diagnosis, ng/ml (IQR)	42.0 (14.1–159.0)	89.0 (12.5–566.0)	0.069
NA	1	2	
**BIOPSY GLEASON SCORE**, ***N*** **(%)**			
<7	13 (16.3%)	11 (13.8%)	
7	32 (40.0%)	27 (33.8%)	
≥8	35 (43.8%)	42 (52.5%)	0.54
NA	9	13	
**CLINICAL T-STAGE**, ***N*** **(%)**			
T1/2	41 (50.6%)	28 (34.1%)	
T3	30 (37.0%)	38 (46.3%)	
T4	10 (12.3%)	16 (19.5%)	0.091
NA	8	11	
**CLINICAL N-STAGE**, ***N*** **(%)**			
N0	64 (79.0%)	55 (64.0%)	
N1	17 (21.0%)	31 (36.0%)	0.031^*^
NA	8	7	
**CLINICAL M-STAGE**, ***N*** **(%)**			
M0	47 (52.8%)	43 (46.2%)	
M1	42 (47.2%)	50 (53.8%)	0.38
**HORMONAL THERAPY**, ***N*** **(%)**			
Combined androgen blockade	49 (55.1%)	60 (64.5%)	
Castration	31 (34.8%)	20 (21.5%)	
Antiandrogen	9 (10.1%)	13 (14.0%)	0.13

* *Statistically significant. IQR, interquartile range; NA, not available*.

## Discussion

Genetic variation in the *MR* gene (Ile180Val) as well as its linked variants such as G215C and Ala241Val was reported to show reduced activity of MR in an *in vitro* assay (15), although the association with serum aldosterone level is controversial (16, 17). In this study, men carrying a homozygous variant with reduced activity of MR showed a worse prognosis when treated with ADT, which is consistent with the pre-clinical finding that decreased MR signaling was associated with increased cellular resistance to hormonal therapy (6). Genetic variations in *MR* may be promising biomarkers of ADT for castration-sensitive prostate cancer, warranting further validation studies.

As well, MR signaling is well known to be associated with hypertension (9, 18). The Flamingham Offspring Study showed that high levels of plasma aldosterone predict the development of hypertension (19). In this study, men comorbid with hypertension that may be associated with higher levels of plasma aldosterone and consequent activated MR signaling showed a better prognosis when treated with ADT, which is consistent with the finding in pre-clinical study that activated MR signaling was associated with vulnerability to antiandrogen therapy (6). However, comorbidity with hypertension was associated with favorable clinicopathological characteristics such as PSA level, Gleason score, and clinical N-stage, but not an independent prognostic factor. This finding suggests that comorbidity with hypertension may be associated with early detection of prostate cancer by regular medical care for hypertension, resulting in lead-time bias of better prognosis.

Previously, variant allele in *MR* gene (rs5522) was associated with a lower risk of hypertension (20). Thus, the finding obtained in this study suggests that individual variations in MR signaling associated with genetic inheritance and comorbidity with hypertension influenced the efficacy of AR-targeting therapeutics. However, this study failed to show this association, which may be derived from the small number of cases, or overlook of comorbidity with hypertension by inadequate definition of hypertension in this study. Thus, a mechanistic interpretation of the relationship of genetic variations in *MR* gene (rs5522) and comorbidity with hypertension in ADT outcome remains unclear.

This study had several limitations including its retrospective design and relatively small sample size. Moreover, comorbidity with hypertension was defined only by regular intake of anti-hypertensive agent, which may lead to overlook of comorbidity with hypertension. However, this exploratory study has shown the possibility of association between prognosis in ADT and genetic variation in *MR* as well as comorbidity with hypertension.

In conclusion, genetic variation in *MR* gene (rs5522) as well as comorbidity with hypertension was associated with prognosis when treated with ADT. This suggests that the individual intensity of MR signaling may be associated with resistance to ADT and a promising biomarker of ADT.

## Author Contributions

MS and NF designed the study, carried out the analysis, analyzed the results, and wrote the manuscript. NF designed and supervised the study, and edited the manuscript. KI, EK, AT, JI, KT, SK, and TU collected the data. ME supervised the study.

### Conflict of Interest Statement

The authors declare that the research was conducted in the absence of any commercial or financial relationships that could be construed as a potential conflict of interest.
